# Effect of Microstructure on the Mechanical Properties of Steel Fiber-Reinforced Recycled Concretes

**DOI:** 10.3390/ma15114018

**Published:** 2022-06-06

**Authors:** Hanquan Yuan, Lihua Zhu, Xiaopeng Wang, Hongtao Yang

**Affiliations:** 1 School of Civil Engineering, Xi’an University of Architecture & Technology, 13 Yanta Road, Xi’an 710055, China; yuanhanquan@126.com (H.Y.); wangxiaopengxa@126.com (X.W.); 2 State Key Laboratory of Green Building in Western China, Xi’an University of Architecture & Technology, 13 Yanta Road, Xi’an 710055, China; 3 Shaanxi Hong Wei Ecological Environmental Protection Technology Co., Xi’an 710100, China; yanghongtaoxa@126.com

**Keywords:** steel fiber-reinforced recycled concrete, macromechanical properties, microstructure, interface transition zone

## Abstract

A steel fiber-reinforced recycled concrete (SFRRC) is a porous material, and its macromechanical properties are affected by its microstructure. To elucidate the change rules and internal mechanisms of the mechanical properties of SFRRCs, the mechanical properties and failure modes of SFRRCs were studied at different water–cement ratio, replacement rate of recycled concrete aggregate (RCA), and steel fiber content. Moreover, the microstructures of the interface transition zones (ITZ) of the SFRRC specimens were tested by scanning electron microscopy and mercury intrusion, and the effect of the microscopic pore structure on the macromechanical properties of SFRRC was analyzed. The research results showed that an appropriate amount of steel fibers could reduce the size and number of cracks in the ITZ and improve the pore structure of an SFRRC. Based on the fractal dimension, porosity and other factors, the quantitative relationship between the macromechanical properties and microscopic pore structure parameters of SFRRCs was established.

## 1. Introduction

Since the beginning of the 21st century, concrete has been the most important material basis for urban construction, and its demand has also been increasing. Currently, the annual output of concrete globally is approximately 4 billion m^3^, and the output of China accounts for approximately 70% of the global total. In urban construction, the demolition of old buildings produces a large amount of construction and demolition waste, which causes environmental pollution [1,2]. Concurrently, the demand for aggregates for concrete production is increasing. Recycled concrete aggregates (RCAs) produced from waste concrete by crushing, screening, and cleaning are used to replace natural concrete aggregates (NCAs), which can effectively solve the dual problems of environment and resources, with very significant economic, social, and environmental benefits [3].

**Figure 1:**
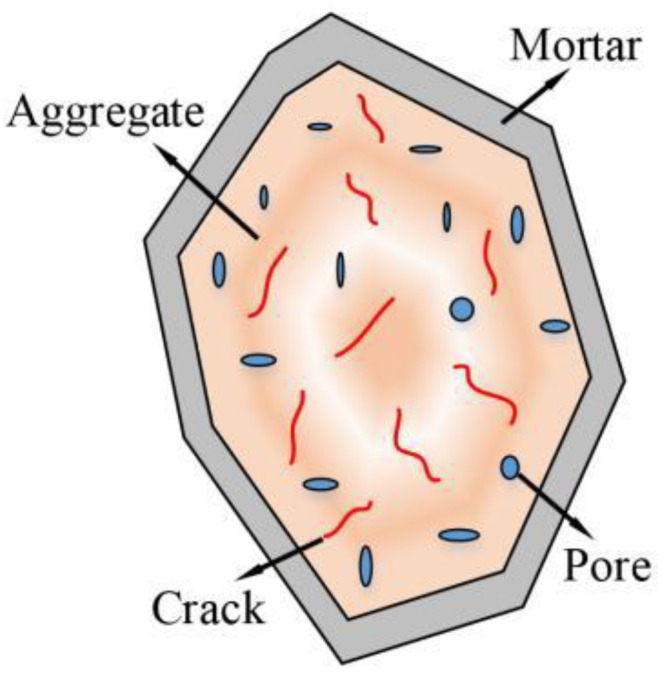
Recycled concrete aggregates.

Research results show that RCAs are damaged during the production process, which produces numerous microcracks inside them [4]. Simultaneously, RCAs feature more edges and corners than NCAs, and hardened cement mortar can become attached to their surfaces, as shown in Figure 1. Because of these factors, RCAs have higher porosity and water absorption than NCAs, and their physical properties are poor [5]. To improve the mechanical properties of recycled aggregate concrete (RAC), scholars usually use concrete-modification technology. These modification techniques mainly include removing the cement mortar on the surface of the RCA (physical method) [6,7,8], filling the pores and cracks inside the RCA with chemical solutions (chemical method) [9,10,11], and doping fibers to improve the pore structure of the interface transition zone (ITZ) [12,13,14]. Compared with the complicated operation process of physical and chemical methods, the method of doping fibers is more convenient.

**Figure 2:**
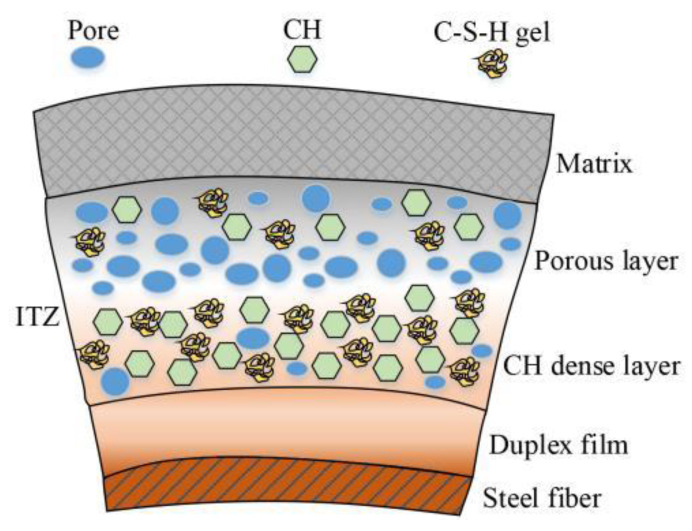
Interface transition zone of SFRRC.

In engineering practice, commonly used fibers are steel fiber (SF) [15], polypropylene fiber [16], copper fiber [17] and basalt fiber [18]. Because SFs can improve and inhibit the development of concrete cracks, improve the quality of RAC, and at the same time, steel fibers can be mass-produced and cheap, the research and application of steel fiber-reinforced concrete has developed rapidly in recent years [19]. According to Bentur, the mechanical properties of steel fiber-reinforced concrete is primarily determined by the bonding properties between the SFs and cement matrix [20]. The ITZ is a weak zone between the SF surfaces and the concrete matrix, as shown in Figure 2. The porosity of the ITZ is relatively high, and its width is approximately 40 µm. The pullout process of the SFs in the matrix is the relative slipping of the SFs caused by the destruction of the structure of the ITZ. Hannawi believes that the SFs have a more hydrophilic surface, and the surrounding cement paste will hydrate and fill the space between the SF and the cement matrix, making its interface transition zone more compact [21]. However, the research on SFRRCs mainly focuses on the macromechanical properties, which are mainly qualitative descriptions. SFRRC is a porous material whose pore structure determines the macromechanical properties. The relationship between the macromechanical properties of SFRRC and the microscopic pore structure has not been discussed in detail, which requires further quantitative research.

Therefore, the main purpose of this study is to explore the relationship between the macromechanical properties of SFRRC and the microscopic pore structure. This work tested the macromechanical properties and microstructure of nine sets of SFRRC specimens with different water–cement ratios, RCAs replacement rates, and SFs content. Finally, a modified formula for the calculation of the strength of SFRRCs based on natural concrete is proposed, and the reasons for the variation of macromechanical properties of SFRRCs from the perspective of microstructure were revealed, and a quantitative relationship between the strength of SFRRCs and microscopic pore structure parameters was proposed.

## 2. Materials and Methods

### 2.1. Materials

**Figure 3:**
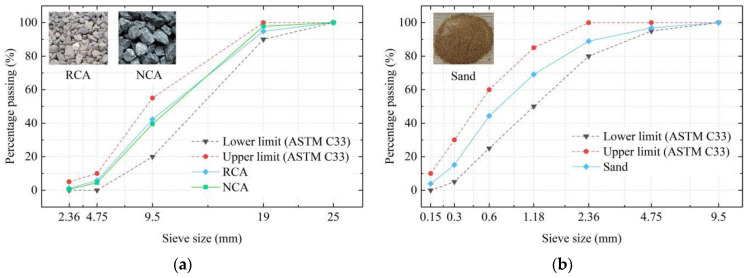
Particle dimension distributions of aggregates: (**a**) Coarse aggregates; (**b**) Fine aggregates.

The coarse aggregates (RCAs and NCAs) used in this study were purchased from Shaanxi Hong Wei Ecological Environmental Protection Technology Co., Ltd. (Xi’an, China), and the fine aggregates were natural river sand from Xi’an, Shaanxi Province. Among them, RCAs mainly come from the waste concrete blocks generated from the demolition of old buildings (20–30 years old) in Xi’an city, the strength of these concrete blocks is about C20 to C40, and the adhered mortar content of RCAs is in the range of 40–45%. The size ranges of the coarse and fine aggregates were 5–20 mm and 0–5 mm, respectively, and the particle dimension distributions of the aggregates are shown in Figure 3. It can be seen that the aggregates satisfied the ASTM-C33 [22] limit requirements. The primary physical properties of the employed RCAs, NCAs, and sand were tested in accordance with the Chinese code GB/T14685-2011 [23]; these are listed in Table 1. The results show that the density of the RCAs is lower than those of the NCAs and that the crushing index and water absorption are higher than those of the NCAs; this is related to the adhesion of the old bonding mortar to the surface of the RCAs and the internal microcracks.

**Figure 4:**
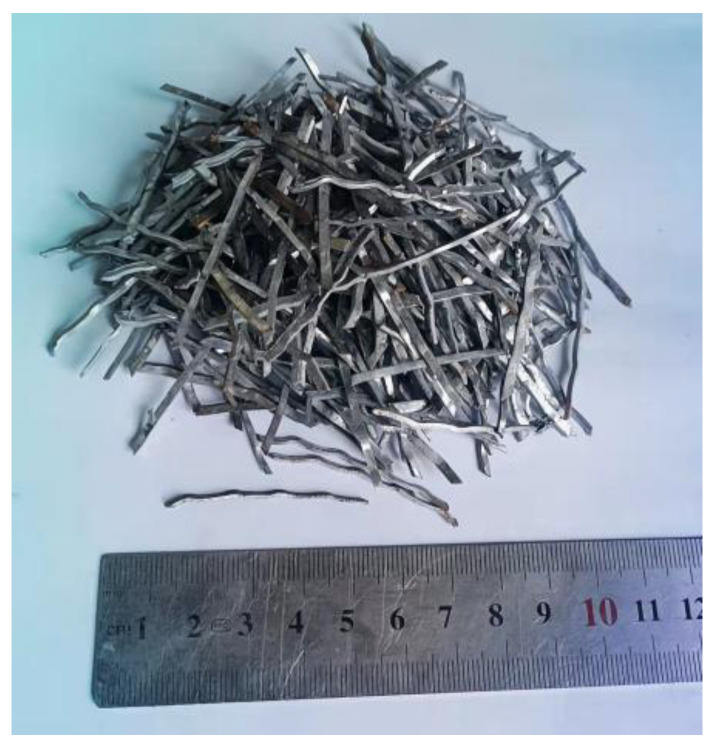
Steel fibers.

The shear-cut type of the SFs used in this study, which are wavy overall, is shown in Figure 4. The length (*l*_f_) of the SFs is 38.0 mm, the equivalent diameter (*d*_f_) is 0.6 mm, the aspect ratio (*l*_f_/*d*_f_) is 63.3, and the tensile strength is 1000 MPa. The cementitious materials used in all the mixtures were cement (P.O42.5), whose physical and chemical properties are listed in Table 2. The water-reducing agent used in this test is polycarboxylic acid high-performance water-reducing agent, the water reduction rate was approximately 25%, and its indexes are shown in Table 3.

### 2.2. Mixture Proportions and Specimen Preparation

In this research, the effects of the water–cement ratio (*w*/*c*), replacement rate of the RCAs (*r_a_*), and SF volume content (*v*_sf_) on the mechanical properties of SFRRCs were studied. To effectively analyze the influence of various factors on the mechanical properties of SFRRCs, orthogonal experiments were conducted, as summarized in Table 4. Based on previous research [24], in these tests, the unit water consumption and the sand rate were set as 160 kg/m^3^ and 0.44, respectively. The mixture proportions for the SFRRC specimens are listed in Table 5.

**Figure 5:**
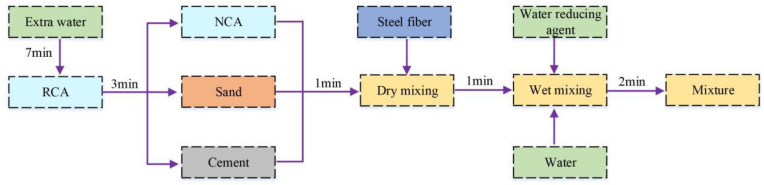
SFRC mixing process.

The mixing process to form SFRRC specimens with improved properties is shown in Figure 5, which can be summarized as the following phases:

Phase I: First, the RCAs and extra water are mixed for 7 min to ensure the RCAs reach a saturated dry surface. Subsequently, they are mixed for 3 min.

Phase II: The NCAs, sand and cement are mixed for 2 min, and the SFs are added in the process of mixing in a manner that they are evenly dispersed in the dry mixture.

Phase III: Finally, water and a water-reducing agent are added to the mixer and stirred for 2 min, and subsequently the mixture is placed into molds.

For each mixture, six cubic and prismatic specimens were prepared for testing the cubic compressive strength (*f*_cu_), splitting tensile strength (*f*_ts_), axial compressive strength (*f*_c_), and elastic modulus (*E*_c_) of the SFRRCs. Their dimensions were 100 mm × 100 mm × 100 mm and 150 mm × 150 mm × 300 mm, respectively.

### 2.3. Test Procedures

#### 2.3.1. Mechanical Properties Test

**Figure 6:**
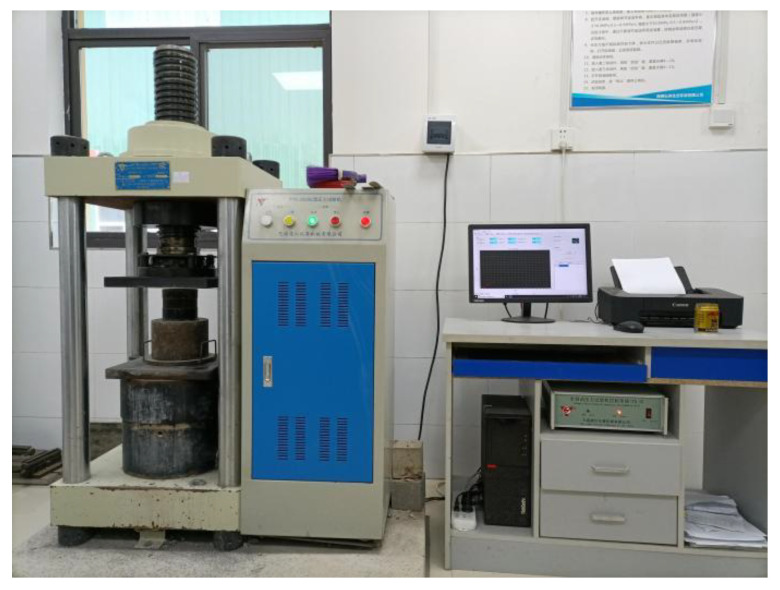
Pressure-testing machine.

A TYE-2000E machine was used for all tests, which is shown in Figure 6. The test method was in accordance with the “Standard for test method of mechanical properties on ordinary concrete” (GB/T50081-2002) [25]. The loading rate of the testing machine was set as 0.5 MPa/s until failure of the cubic and prism specimens occurred to obtain *f*_cu_ and *f*_c_. When testing *f*_ts_, the loading rate was 0.05 MPa/s, and *f*_ts_ is calculated using Equation (1).

(1) $$f_{\mathrm{ts}}=\frac{2F}{\pi A}=0.637\frac{F}{A}$$

where *F* is the ultimate load and *A* is the splitting surface area. Because the cube test block was a nonstandard test block, *f*_ts_ was multiplied by a conversion factor of 0.85 [25].

**Figure 7:**
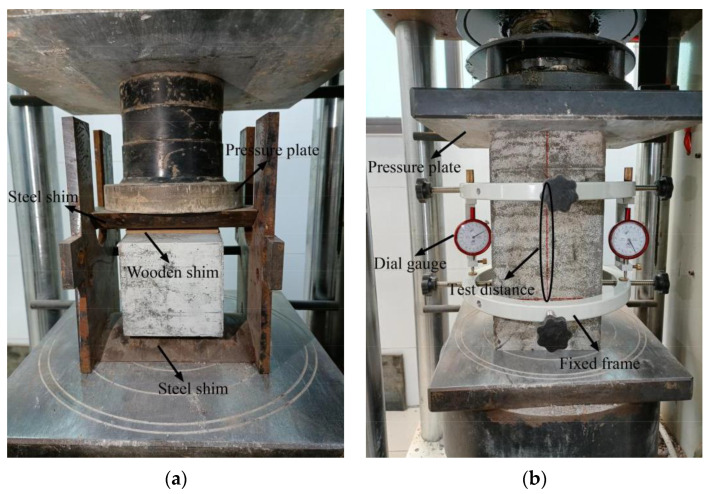
Mechanical property test setup: (**a**) Splitting tensile test; (**b**) Elastic modulus test.

Figure 7 depicts the mechanical property test setup. In the splitting tensile test, a circular arc steel shim and a wooden shim were placed between the pressure plate and the specimen. In the elastic modulus test, the test distance was 150 mm in the mid of the prism sample, and two dial gauges were placed on both sides of the test piece to measure the displacement.

The specimen needs to be preloaded to ensure axial loading. First, loading from 0 to 0.5 MPa (*F*_0_) is applied, which is followed by a constant load for 60 s. Subsequently, a load equal to the stress of *f*_c_/3 load (*F_a_*) is applied, followed again by a constant load for 60 s to ensure that the deformation difference between the two sides is less than 20%. This loop is repeated thrice, following which the preloading ends. After unloading the load, the load is increased from 0 to *F*_0_, the deformation value, *L*_0_, is recorded; subsequently a load from *F*_0_ to *F_a_* is added, and the deformation value, *L_a_*, is recorded. Finally, the deformation measuring instrument is removed, and the specimen is loaded to failure. The difference between the failure load and the previously measured axial compressive strength should not exceed 20%. The elastic modulus is calculated using Equation (2).

(2) $$E_{c}=\frac{F_{a}-F_{0}}{A}\times \frac{L}{L_{a}-L_{0}}$$

where *E*_c_ is the elastic modulus, *F_a_* is taken as *f*_c_/3, *F*_0_ is taken as 0.5 MPa, *A* is the pressure-bearing area of the specimen, *L* is the test distance of the specimen, and *L_a_* and *L*_0_ are the average deformation values on the two sides when the loads are *F_a_* and *F*_0_, respectively.

#### 2.3.2. Microstructure Test

**Figure 8:**
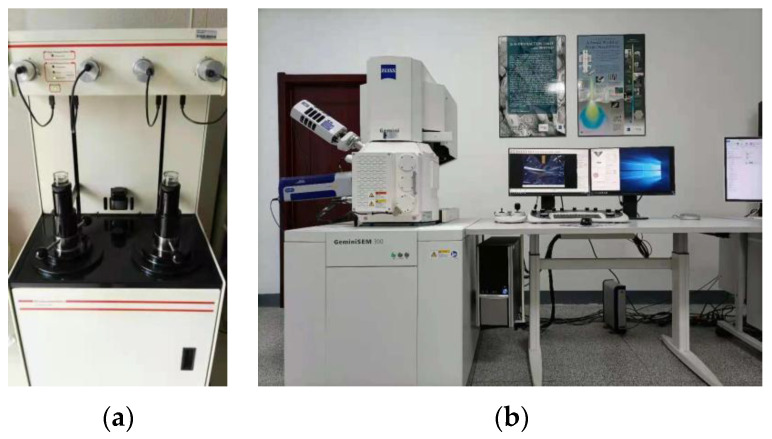
Test instruments: (**a**) Mercury porosimeter; (**b**) Scanning electron microscope.

Concrete is a type of porous material, and it has various pore shapes. Mercury intrusion porosimetry (MIP) is frequently used to analyze the microscopic pore structure of materials. A scanning electron microscope has a high resolution, magnification, and large depth of field, and it can analyze the characteristics of a material at the nanometer level. Thus, the microscopic morphologies of the SFRRC specimens were analyzed using scanning electron microscopy (SEM), and their pore structures were analyzed via MIP. In this research, the SEM experiments employed a Gemini SEM300 scanning electron microscope (Oberkochen, Germany) with a resolution of 0.8 nm and an acceleration voltage of 30 KV. The MIP experiments were conducted using an American AutoPore IV 9500 (Norcross, GA, USA), and the pressure range was 0.003–300 MPa. The test instruments are shown in Figure 8.

## 3. Results and Discussion

### 3.1. Failure Mode of SFRRC

Before the tests, the specimens are placed in a curing chamber for 28 days. The temperature and relative humidity of the curing chamber are, respectively, 20 ± 2 °C and not less than 95%.

**Figure 9:**
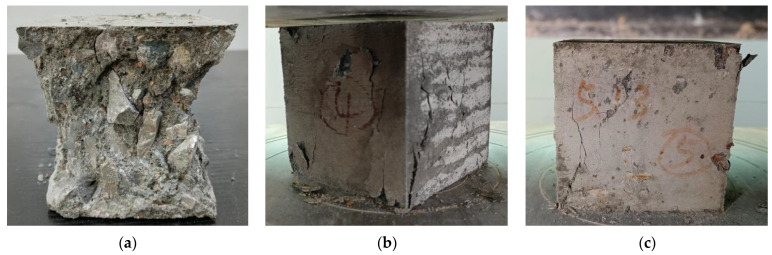
Failure modes in cubic compressive tests: (**a**) *v*_sf_ = 0%; (**b**) *v*_sf_ = 1%; (**c**) *v*_sf_ = 2%.

**Figure 10:**
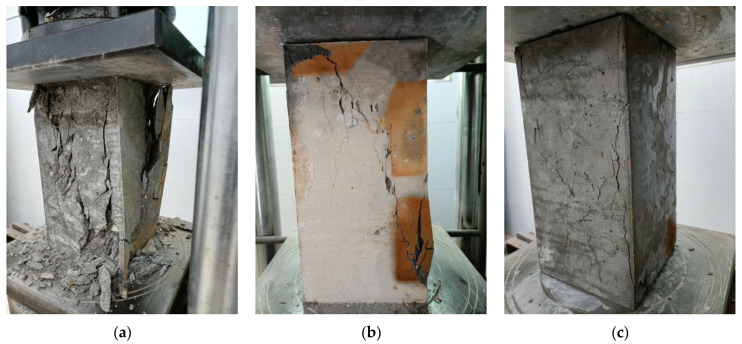
Failure modes in axial compression tests: (**a**) *v*_sf_ = 0%; (**b**) *v*_sf_ = 1%; (**c**) *v*_sf_ = 2%.

In the cubic compressive tests, a specimen without SFs undergoes compression deformation and transverse elongation deformation during compression. At the initial stages of loading, microcracks appear on the surface of the concrete and gradually develop into edges and corners. With an increase in the load, the rapid development of microcracks occurs, expanding both the concrete inside and the surface of the specimen, with the latter severely falling off. Finally, the concrete is crushed, and the failure mode exhibits a wedge shape on the surface of the specimen, which is understandably brittle failure. During the compressive failure process, cracks appear later in the specimens mixed with SFs than in the specimens without SFs. The cracks are significantly small in the early stages, and the microcracks gradually develop and become larger in the later stages, with reduced peeling of the surface of the test specimen. Until the end of the loading of a specimen with SFs, the specimen does not undergo wedge failure and maintains good integrity, showing remarkable plastic failure. A large amount of SFs implies fewer cracks, less shedding, and good integrity. It is found that the addition of SFs improves the failure mode of the concrete and transforms it from brittle failure to plastic failure. The failure modes in the axial compressive strength tests are basically the same as those in the cubic compressive strength tests. The typical failure modes in the cubic and prismatic axial compressive tests of the SFRRC specimens are shown in Figure 9 and Figure 10, respectively. The failure modes of the SFRRCs with different replacement ratios for the RCAs and water-to-binder ratios are similar.

**Figure 11:**
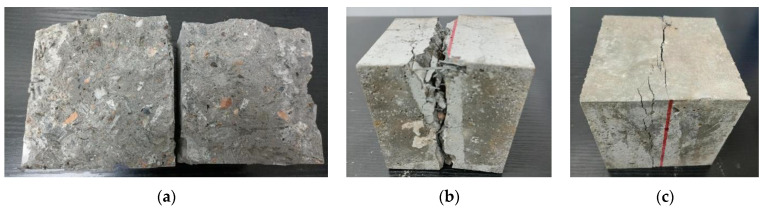
Failure modes in splitting tensile tests: (**a**) *v*_sf_ = 0%; (**b**) *v*_sf_ = 1%; (**c**) *v*_sf_ = 2%.

In the splitting tensile tests, a specimen without SFs starts to show microcracks along the shims as the load is first increased. When the ultimate splitting strength is reached, the cracks rapidly penetrate the bottom and top surfaces of the specimen, at which time the load drops sharply, and the specimen is split into two parts. The damage is heard with a notable ringing sound, which belongs to brittle failure. The failure surface of the specimen shows that the fracture occurs mainly at the intersection of the aggregates and the cement mortar, and the aggregates themselves are not pulled off. The failure mode of a specimen changes significantly after the addition of SFs. As the load is increased, the cracks on the surface of the concrete gradually increase, a part of the concrete begins to fall off, and cracks develop through the bottom and top surfaces. However, the specimen is not split into two parts owing to the connection of the SFs between the cracks. The SFRRC has good integrity, and its failure mode belongs to plastic failure. The results show that the admixture of SFs effectively inhibits the development of cracks. The SFs consumes a large part of the energy, and cracks are produced mainly on the surface of the specimen during the failure process and the concrete is not severely peeled off and broken, and these phenomena become more significant with the increase in the SF admixture. The typical failure modes of the SFRRC specimens in the splitting tensile tests are shown in Figure 11.

### 3.2. Mechanical Properties of SFRRC

Loads are applied to the specimens by a pressure-testing machine, and the *f*_cu_, *f*_ts_, *f*_c_, and *E*_c_ of each group of specimens are obtained, as summarized in Table 6. The values in Table 6 are the averages of the test results of three specimens in each group.

**Figure 12:**
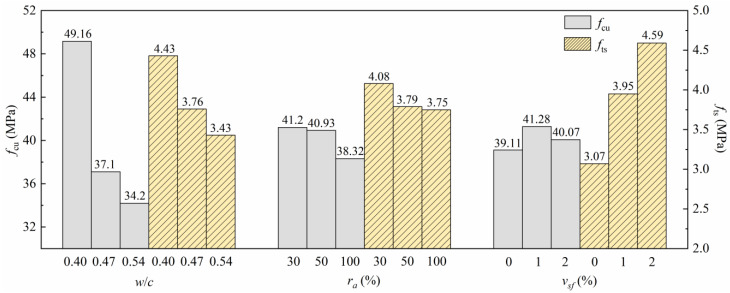
Relationships between various factors and mechanical properties.

It can be found that there is a significant linear relationship between *f*_cu_ and *f*_c_, with the ratio fluctuating at approximately 0.73, which is similar to that of natural concrete. The ratio of *f*_ts_ to *f*_c_ significantly fluctuates, which is mainly due to the large influence of the SFs on the splitting tensile behavior of the concrete. *f*_cu_ and *f*_ts_ are the main mechanical properties of concrete. The results in Table 6 are processed according to the orthogonal test analysis method, and the mean response analysis of the mechanical properties is shown in Figure 12.

It was found that *f*_cu_ decreases with the increase in *w*/*c* and *r_a_*, which is consistent with the research results of [26]. The *f*_cu_ and *f*_ts_ of the SFRRCs only decreased by 6.38% and 1.06% when *r_a_* increased from 50% to 100%, so it is recommended that *r_a_* be increased to achieve more consumption of recycled aggregates in actual engineering. The SFs had a particularly significant influence on *f*_ts_, when the SFs volume content is 1% and 2%, the *f*_ts_ increases by 28.66% and 49.51%. At the same time, the *f*_cu_ increased by 5.55% and 2.45%, indicating that too much SFs dosing will instead reduce the *f*_cu_ of SFRRC, after considering *f*_cu_ simultaneously, it is recommended that the amount of SFs should not be extremely large. Since SFs have a significant impact on the mechanical properties of recycled concrete, the microstructure of SFRRC will be analyzed in detail in the follow-up, and the reasons for the change in macromechanical properties will be explained through the microstructure.

### 3.3. Strength Calculation Model

Tensile strength is an important property for reflecting the cracking resistance of concrete, and most scholars advocate the use of splitting tensile tests of cylinders or cubes to indirectly determine the tensile strength of concrete [27]. Elastic modulus (*E*_c_) reflects the relationship between the stress and deformation of a material in elastic state, and it is a necessary parameter for calculating deformation in concrete structure design [28]. The results of numerous studies have shown that there is a relationship between the *f*_ts_, *E*_c_, and *f*_cu_ of concrete [29,30]. In this study, *f*_ts_ and *E*_c_ are evaluated according to Chinese code GB50010-2010 [31] as follows:

(3) $$f_{\mathrm{ts}}=0.19f_{cu}^{0.75}$$

(4) $$E_{c}=\frac{10^{5}}{2.2+34.7/f_{\mathrm{cu}}}$$

Similar formulas are available in the code, ACI318-14 [32], as expressed in Equations (5) and (6).

(5) $$f_{\mathrm{ts}}=0.55f_{cu}^{0.5}$$

(6) $$E_{c}=4700\sqrt{f_{\mathrm{cu}}}$$

**Figure 13:**
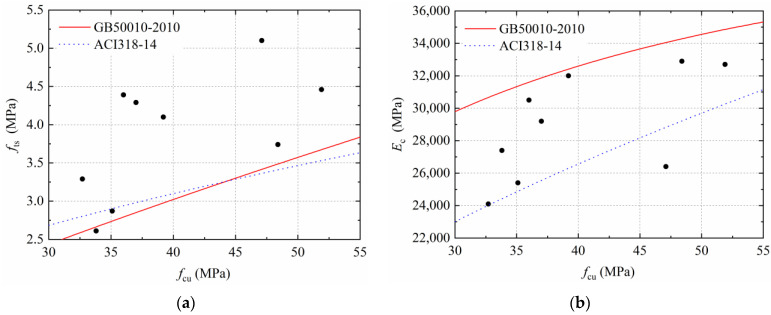
Comparisons between tests and standards: (**a**) Relationship between *f*_cu_ and *f*_ts_; (**b**) Relationship between *f*_cu_ and *E*_c_.

The calculation results of the above formula are compared with the test results of this study, and the connections between *f*_ts_, *E*_c_, and *f*_cu_ are drawn as scatter points in Figure 13. It can be seen that the test values of *f*_ts_ are larger than the standard calculated values, mainly because of the significant enhancement in *f*_ts_ caused by the SFs. In general, SFs enhance the *E*_c_ of concrete, whereas the RAC shows a suppressive effect, and a combined effect of the two factors in a test value of *E*_c_ is a large deviation from the standard calculated values. Therefore, the calculation formula for natural concrete is unsuitable for the SFRRCs.

According to the formula in the standard, *f*_ts_ and *E*_c_ are still calculated from *f*_cu_. Considering the influence of different factors in the orthogonal tests on the *f*_cu_ of the SFRRCs, multiple parameters need to be defined to predict *f*_cu_. Assuming that there are linear relationships between the *f*_cu_ of SFRRCs and the water–cement ratio, replacement rate of the RCAs, and SF volume content, a calculation model for SFRRCs based on the following formula is proposed:

(7) $$f_{\mathrm{cu}}=b_{1}+b_{2}\left(w/c\right)+b_{3}r_{a}+b_{4}\lambda_{m}$$

(8) $$f_{\mathrm{ts}}=c_{1}f_{cu}^{c_{2}}\left(1+c_{3}\lambda_{m}\right)\left(1+c_{4}r_{a}\right)$$

(9) $$E_{c}=\frac{10^{5}}{2.2+34.7/f_{\mathrm{cu}}}\left(1+d_{1}\lambda_{m}\right)\left(1+d_{2}r_{a}\right)$$

where *b_i_* (I = 1–4) are the regression coefficients of the compressive strength, *w*/*c* is the water–cement ratio, *r_a_* is the replacement rate of the RCAs, *λ_m_* is the product of the SF volume content and the aspect ratio of the SFs, *c_i_* (I = 1–4) are the regression coefficients of the splitting tensile strength, and *d_i_* (I = 1, 2) are the regression coefficients of the elastic modulus.

**Figure 14:**
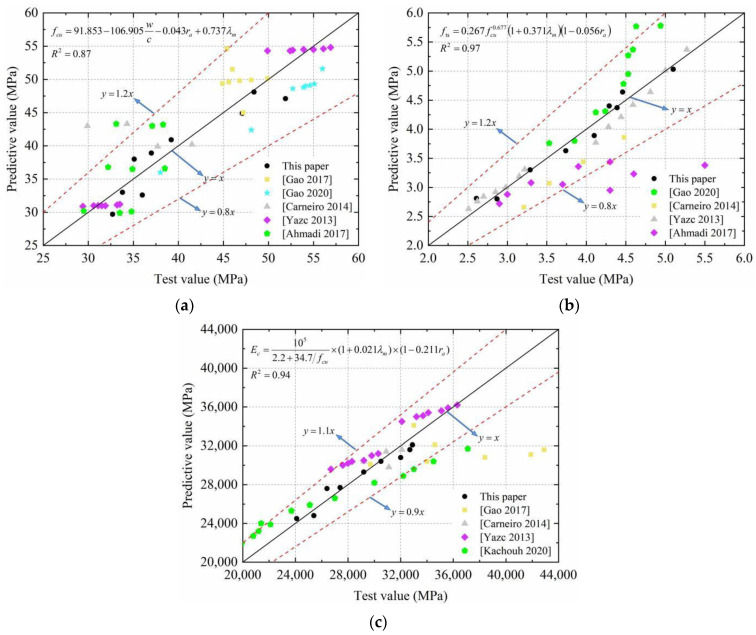
Accuracy of prediction model for mechanical properties of SFRRC: (**a**) Compressive strength; (**b**) Splitting tensile strength; (**c**) Elastic modulus.

The formulas for the *f*_cu_, *f*_ts_, and *E*_c_ of SFRRCs were established by regression analysis. As expected, Figure 14 shows that the fitting coefficients are sufficient for satisfying the requirement. To verify the reliability of Equations (7)–(9), the data of Gao [33,34], Carneiro [35], Yazc [36], Ahmadi [37], and Kachouh [38] were used to compare the calculation results of the formula with the test results. The results are listed in Table 7. It can be found that the predicted values are very close to the actual values. The maximum error of *f*_cu_ and *f*_ts_ is approximately 20%, the average error is approximately 5%, the maximum error of *E*_c_ is approximately 10%, and the average error is approximately 2%. Consequently, the modified formulas can be applied to predict the *f*_cu_, *f*_ts_, and *E*_c_ of SFRRCs with satisfactory accuracy.

### 3.4. Morphology of ITZ

**Figure 15:**
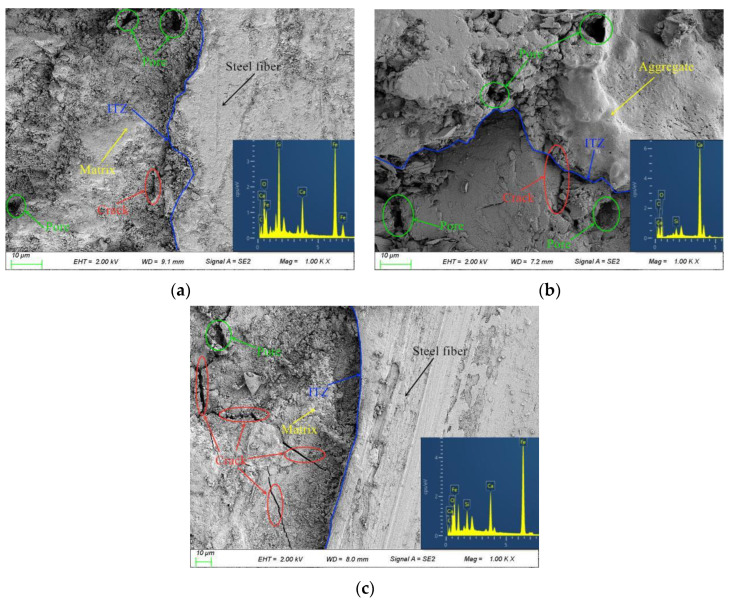
Morphologies of ITZs: (**a**) *v*_sf_ = 1%; (**b**) *v*_sf_ = 0%; (**c**) *v*_sf_ = 2%.

Concrete is a composite material consisting of three parts: aggregates, mortar, and aggregate–mortar ITZ, where the ITZ is the weakest region in concrete and has a significant impact on the strength of concrete. Figure 15 shows the morphology of the ITZs of the SFRRCs with different SF volume contents.

From Figure 15a, it can be found that there is a microcrack between the SFs and the cement matrix. The SFs are pulled out from the cement matrix mainly because the ITZ structure is damaged, which eventually leads to the relative sliding of the SFs and the matrix. From Figure 15b,c, it can be found that the holes and cracks near the ITZ are significantly reduced at 1% SF doping compared to that in the specimens without SFs. However, the number and size of the cracks near the ITZ increases significantly when the SF dosing is 2%.

### 3.5. Microstructural Analysis of the SFRRC

**Figure 16:**
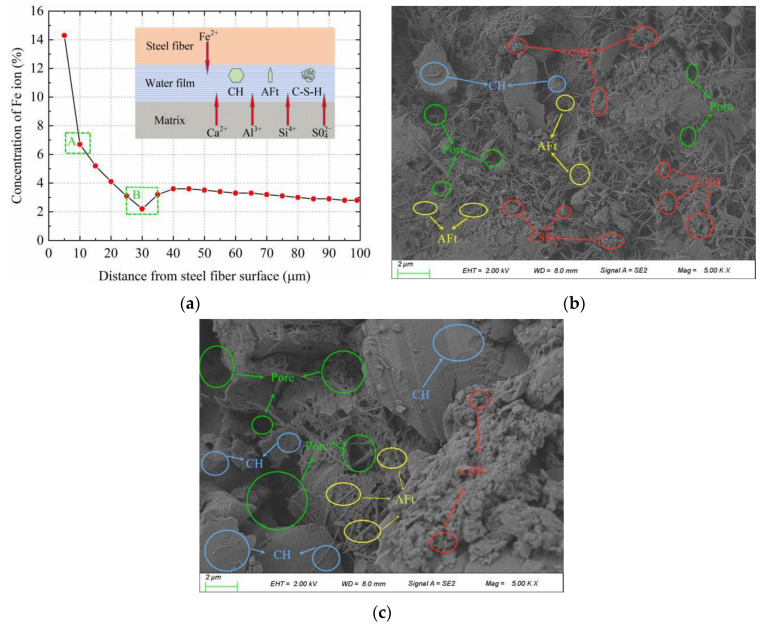
Micromorphology of SFRRC: (**a**) Element fraction by EDXA; (**b**) A area; (**c**) B area.

For the aggregate ITZ, when cement is dissolved in water, the dissolved ions diffuse and enter the water film. The active order of ions is Na^+^ > K^+^ > SO_4_^2−^ > Al^3+^ > Ca^2+^ > Si^4+^, and the first products to be formed in the water film are hexagonal-shaped calcium hydroxide (CH) and needle-shaped ettringite (AFt). Compared to the cement matrix, the water–cement ratio in the ITZ is higher, and its ion saturation is lower. Thus, CH and AFt can grow freely and oriented on the aggregate surface without restriction. This prevents the calcium silicate hydrate (CSH) gel from contacting the aggregates and increases the porosity close to the ITZ. As the ion concentration in the ITZ decreases, the amount of the CSH gel is reduced, which eventually leads to the formation of a loose network structure in the ITZ. Therefore, the weakest position in the ITZ of the aggregates is that close to the surface of the aggregates. Similarly, the mechanical properties of an SFRRC also strongly depend on the microstructure of the ITZ, i.e., the area where the SFs are in contact with the cement matrix. Results of the SEM with energy-dispersive X-ray analysis (SEM-EDXA) for an SF-cement matrix-based ITZ are shown in Figure 16. Figure 16b,c show the effect of area A (25–35 µm from the SF surface) and area B (5–15 µm from the SF surface) magnified by 5000 times, respectively.

It can be seen that, as the distance from the surfaces of the SFs increases, the concentration of Fe^2+^ ions first decreases and subsequently increases. At 25–35 µm from the surfaces of the SFs, the Fe^2+^ ion concentration is close to the lowest value. After 40 µm, the Fe^2+^ ion concentration begins to level off, and at 100 µm, it is the inherent Fe^2+^ ion concentration of the cement itself. The results show that a part of the Fe^2+^ ions can be dissolved from the surfaces of the SFs, which is different from an ordinary aggregate interface. This part of the Fe^2+^ ions enters the water film, and the superposition of the ions dissolved in the cement and the ions dissolved from the SFs makes the concentration of ions in the interface higher than that in the ordinary aggregate interface. Fe^2+^ ions can reduce some of the adverse effects of the water film, and more CSH is formed close to the SFs. This increases the probability of the CSH contacting the SFs and causes it to fill the porous structure close to the surface of the SFs, therefore enhancing the compactness of the structure. The Fe^2+^ ion concentration is lower in the A area slightly further from the fiber surface, and the needle-shaped AFt and hexagonal-shaped CH mainly accumulated in the area. Owing to the influence of solid particle shape, the sparse accumulation of particles around the side wall produces numerous pores, resulting in the formation of a loose structure in the ITZ around the aggregates, which can be considered to be a weaker area in the SF–matrix. However, excessive SFs can have a detrimental effect on the concrete, which requires a more detailed analysis of the microscopic pore structure parameters.

## 4. Internal Mechanisms of the Mechanical Properties in SFRRC

### 4.1. Microscopic Pore Structure Analysis

**Figure 17:**
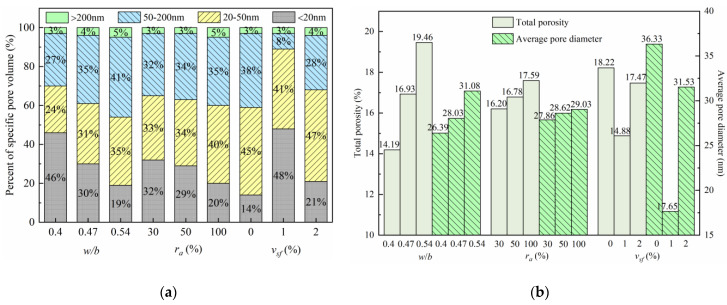
Pore characteristic parameters: (**a**) Pore size distribution; (**b**) Total porosity and pore diameter.

The microscopic pore structures of the SFRRCs were determined using the MIP method, and the pore structure parameters of each specimen are listed in Table 8 and shown in Figure 17. The pore structures can be divided into four levels according to the pore size [39]: harmless pores (diameter < 20 nm), less-harmful pores (diameter 20–50 nm), harmful pores (diameter 50–200 nm), and more-harmful pores (diameter > 200 nm).

From Table 8 and Figure 17, it can be found that the water–cement ratio, replacement rate of the RCAs, and SFs have little effect on the content of the harmful pores in the SFRRCs. With the increase in the water-binder ratio and the RCA replacement rate, the porosity and volume percentages of the less-harmful, harmful, and more-harmful pores of the specimens increase, whereas the volume percentage of the harmless pores decreases. With the increase in the SF volume content, the porosity and pore size of the SFRRCs show first a decreasing and subsequently an increasing trend. When the SF volume content is 1%, the total porosity and average pore diameter of the SFRRCs are the smallest, which are 18.33% and 51.42% lower than those of the specimens without SFs, respectively. When the SF volume content is 2%, all pore structure parameters of the SFRRCs show an increasing trend. Compared to the SFRRC with 1% SF volume content, the total porosity and pore size of the former increase by 17.41% and 78.64%, respectively. The results show that mixing an appropriate amount of the SFs into the concrete can effectively prevent the generation of the concrete microcracks caused by water loss and reduce the size and number of microcracks. These therefore reduce the total porosity of concrete and make the distribution of the pore structure more reasonable. When the amount of the SFs is extremely large, their agglomeration occurs in the concrete, resulting in an uneven distribution of the pore structure, and increasing the total porosity and pore size of the concrete. It is generally believed that the *f*_cu_ of an SFRRC mainly depends on the compactness of its matrix, and the *f*_ts_ of an SFRRC mainly depends on the bonding strength of the SF–matrix interface. The SFs can bond with the matrix well and have the effect of strengthening and toughening the concrete; therefore, the *f*_ts_ of the concrete is significantly improved with the increase in the SFs. However, excessive addition of the SFs adversely affects the pore structure of the concrete, resulting in a decrease in *f*_cu_.

### 4.2. Relationship between Strength and Pore Structure of SFRRCs

**Figure 18:**
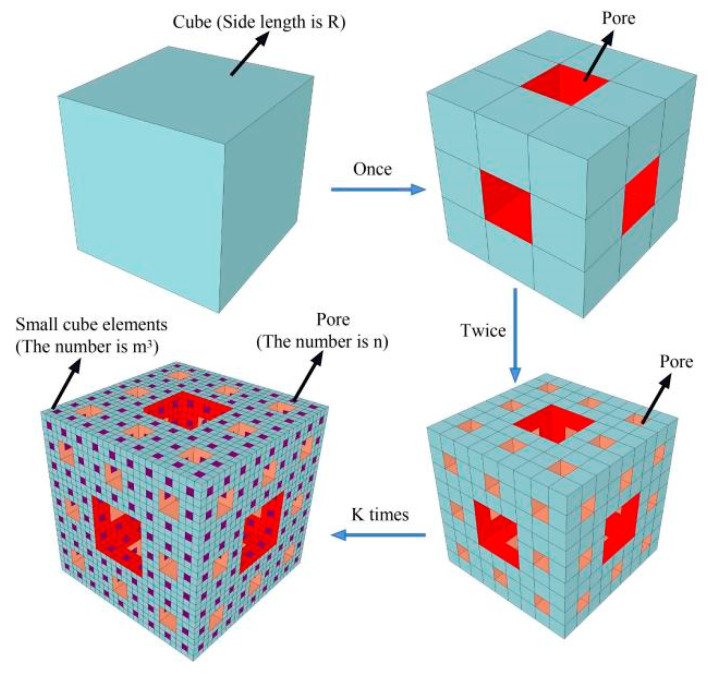
Menger sponge model.

The static pore structure of concrete as a porous medium is generally analyzed using the Menger sponge model, and this method has been recognized by many researchers [40]. As shown in Figure 18, each side of the initial element with side length *R* is equally divided into m parts, following which m^3^-small cube elements are obtained and n small cube elements are randomly eliminated. After k times, the number of remaining cubes is (*m*^3^ − *n*)*^k^* and the side length of the smallest cube unit is *r_k_* = *R*/*m^k^*. The fractal dimension, *D_m_*, of the sponge model is calculated using the box-counting method, as expressed below:

(10) $$D_{m}=\underset{k\to +\infty }{\lim}\frac{\ln{\left(m^{3}-n\right)}^{k}}{\ln\left(m^{k}/R\right)}=\ln\left(m^{3}-n\right)/\ln\;m$$

According to the fractal theory, the remaining cube volume, *V*_s_, of the sponge model has a power relationship with the length, *r_k_*, of the smallest cube elements, and their derivatives can be obtained as follows:

(11) $$\frac{\ln\;V_{s}}{\ln\;r_{k}}=\frac{\ln\;[{\left(m^{3}-n\right)}^{k}\times {\left(R/m^{k}\right)}^{3}]}{\ln\;(R/m^{k})}\propto (3-D_{m})$$

The remaining cube volume of the sponge model is:

(12) $$V_{s}\propto r_{k}{}^{3-D_{m}}$$

The pore volume of the remaining cube, *V_p_*, is:

(13) $$V_{p}=R^{3}-V_{s}$$

Based on Equations (12) and (13), the following formula is obtained:

(14) $$\mathrm{lg}(-dV_{p}/dr_{k})\propto (2-D_{m})\mathrm{lg}\;r_{k}$$

**Figure 19:**
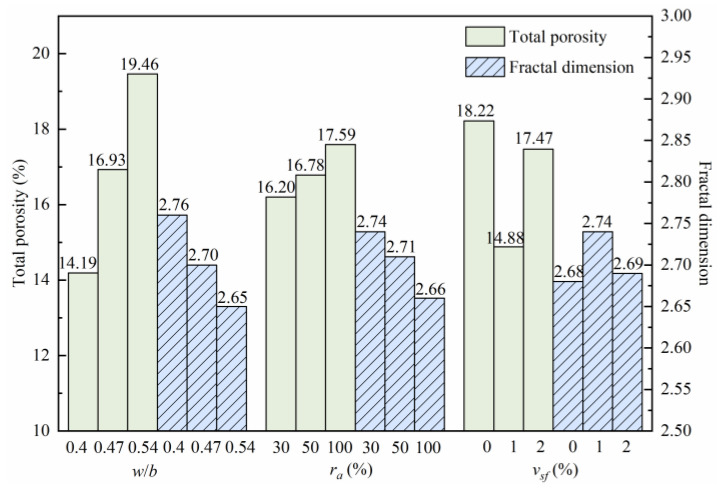
Total porosity and fractal dimension.

It can be seen from Equation (14) that the logarithm of −*dV_p_*/*dr_k_* and *r_k_* have a linear relationship, and the fractal dimension, *D_m_*, can be obtained by fitting of the curve, and the result is shown in Figure 19.

It can be seen from Figure 19 that the variation law of the total porosity of the SFRRCs is opposite to the variation law of the fractal dimension, which indicates that porosity can reflect the variations in the pore structure composition and distribution in the concrete to a certain extent. A large fractal dimension implies a highly complex pore structure and high composition in the concrete, and a high tortuosity of the capillary pores.

**Figure 20:**
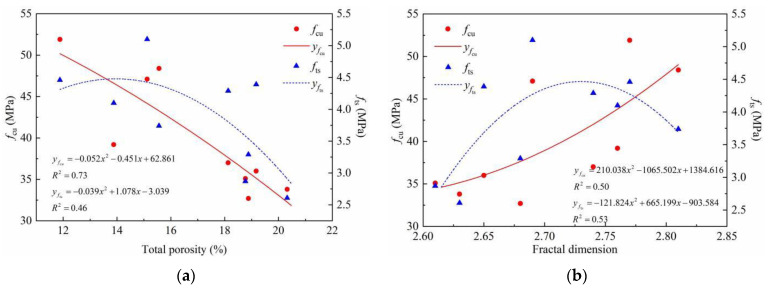
Fitting of SFRRC strength and pore characteristic parameters: (**a**) Total porosity fitting curve; (**b**) Fractal dimension fitting curve.

*f*_cu_ and *f*_ts_ are the most basic mechanical indicators of the macroscopic properties of concrete, and the magnitude of the strength is closely related to the microscopic pore structure. Research results show that the mechanical strength of concrete is related to its porosity [41], pore size distribution, and fractal dimension, and researchers commonly use porosity or fractal dimension to calculate the strength of concrete. The results of the quadratic polynomial fitting of the relations between the porosity, fractal dimension, and strength of the concrete are shown in Figure 20.

It can be found that the fitting between the concrete strength and the porosity or the fractal dimension needs to be improved. To obtain a higher precision calculation model, it is necessary to consider the effects of both the porosity and fractal dimension, and the influence coefficients of the SF volume content and the replacement rate of the RCAs should be added. Based on the above analysis results, a calculation model of SFRRC strength based on the following formula is proposed:

(15) $$f=(e_{1}P_{t}^{2}+e_{2}D_{m}^{2}+e_{3}P_{t}D_{m}+e_{4}P_{t}+e_{5}D_{m}+e_{6})\left(1+e_{7}\lambda_{m}\right)\left(1+e_{8}r_{a}\right)$$

where *f* is the SFRRC strength, *e_i_* (I = 1–8) are the regression coefficients of strength, *D_m_* is the fractal dimension, *λ_m_* is the product of the SF volume content and the aspect ratio of the SFs, and *r_a_* is the replacement rate of the RCAs.

**Figure 21:**
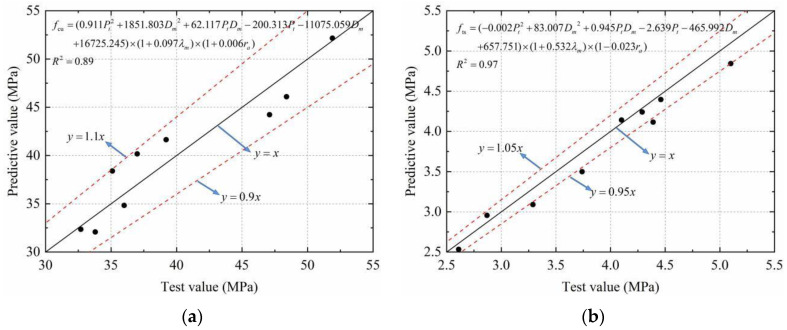
Multiple regression calculation of SFRRC strength: (**a**) Compressive strength; (**b**) Splitting tensile strength.

Based on Equation (15), the relationships between the concrete strength and the pore structure parameters, SF volume content, and RCA ratio were obtained by multiple regression analysis, and *f*_cu_ and *f*_ts_ are calculated as shown in Figure 21. It can be found that the r-squared of the multifactor calculation models for *f*_cu_ and *f*_ts_ are 0.89 and 0.97, respectively. Moreover, the maximum errors of the compressive strength and splitting tensile strength do not exceed 10% and 5%, respectively, indicating that the regression effect is significant. Therefore, the calculation model results are in good agreement with the experimental results and can accurately describe the quantitative relationships between the macromechanical properties of SFRRCs and the microscopic pore structure parameters.

## 5. Conclusions

This study investigated the mechanical properties of SFRRCs with different water–cement ratios, RCA replacement rates, and SF volume contents by orthogonal tests. Combined with an SFRRC microstructure analysis, the following conclusions were drawn:

- The SFs can inhibit the development of SFRRC cracks, increase the strength and ductility of the SFRRC, and significantly improve the failure mode of the SFRRC, changing it from brittle failure to plastic failure.
- With the increase in the SF volume content, the *f*_ts_ of the SFRRCs increase significantly. When the SFs volume content is 1% and 2%, the *f*_ts_ increases by 28.66% and 49.51%. At the same time, the *f*_cu_ increased by 5.55% and 2.45%, indicating that too much SFs dosing will instead reduce the *f*_cu_ of SFRRC, which is related to the microscopic pore structure of SFRRCs.
- The formula for calculating concrete strength in the code is not applicable to SFRRCs. By a multifactor analysis, a mechanical calculation model for SFRRCs is obtained using Equations (7)–(9), and the calculation and test results have a high degree of fit.
- The Fe^2+^ ions dissolved on the surface of the SFs can improve the structure of the ITZ and increase the bonding strength of the interface. The weak region of the SF–matrix ITZ is approximately 25–35 µm from the SF surfaces, which is related to the concentration of Fe^2+^ ions.
- When the SF volume content is 1%, the total porosity and average pore diameter of the SFRRCs are 18.33% and 51.42% lower than those of the specimens without SFs. Compared to the SFRRC with 1% SF volume content, the total porosity and pore size of SFRRCs increased by 17.41% and 78.64%, respectively, when the SF content was 2%. This phenomenon is because adding the appropriate amount of SFs reduces the size and number of cracks in the ITZ and makes the pore structure distributions of the SFRRCs more reasonable. An excessive amount of SFs causes an uneven distribution of the pore structure, increasing the total pore space and the pore size, therefore reducing the compressive strength of the SFRRCs.
- It is inaccurate to use the porosity or the fractal dimension alone to calculate the strength of an SFRRC. The regression of a multifactor strength calculation model using the fractal dimension, porosity, SF volume content, and RCA replacement rate is significant, and the model can accurately describe the quantitative relationships between the strength of the SFRRCs and the pore structure parameters.

## Figures and Tables

**Table 1:** Physical properties of RCAs, NCAs, and sand.

Properties	RCA		NCA		Sand
Particle size (mm)	5–10	10–20	5–10	10–20	0–5
Apparent density (kg/m^3^)	2640	2650	2810	2820	2620
Bulk Density (kg/m^3^)	1290	1330	1510	1550	1520
Water absorption (%)	7.3	5.6	0.8	0.3	0.7
Crushing index (%)	—	13.5	—	8.1	—
Fineness modulus	—	—	—	—	2.76

**Table 2:** Physical and Chemical properties of cement.

Setting Time (min)		Chemical Composition (%)							Strength (MPa)			
									Compressive Strength		Flexural Strength	
Initial Set	Final Set	CaO	SiO_2_	Al_2_O_3_	Fe_2_O_3_	S0_3_	MgO	K_2_O	3d	28d	3d	28d
180	280	60.3	22.5	7.2	3.6	2.1	2.3	0.8	39.1	50.9	6.5	8.1

**Table 3:** Physical and chemical compositions of water-reducing agent.

Density (g/cm^3^)	Solid Content (%)	Chloride Ion Content (%)	Alkali Content (%)	pH Value	Water Reduction Rate (%)
1.062	23.7	0	1.13	6.5	25

**Table 4:** Orthogonal test factors and levels of SFRC.

Levels	Factors		
	*w*/*c* (A)	*r_a_* (B)	*v*_sf_ (C)
1	0.4	30%	0%
2	0.47	50%	1%
3	0.54	100%	2%

Note: *w*/*c*: water–cement ratio; *r_a_*: replacement ratio of RCAs; *v*_sf_: SF volume content.

**Table 5:** Mixture proportions of specimens (kg/m^3^).

Groups	Orthogonal Design	Water	Cement	Sand	NCA	RCA	Steel Fiber	Extra Water	Water-Reducing Agent
SFRRC-1	A1B1C1D1	160	400	788	702	301	0	18	4
SFRRC-2	A1B2C2D2	160	400	753	479	479	78	29	4
SFRRC-3	A1B3C3D3	160	400	719	0	915	156	56	4
SFRRC-4	A2B1C2D3	160	340	779	694	298	78	18	3.4
SFRRC-5	A2B2C3D1	160	340	745	474	474	156	29	3.4
SFRRC-6	A2B3C1D2	160	340	814	0	1036	0	63	3.4
SFRRC-7	A3B1C3D2	160	296	765	681	292	156	18	2.96
SFRRC-8	A3B2C1D3	160	296	833	530	530	0	32	2.96
SFRRC-9	A3B3C2D1	160	296	799	0	1017	78	62	2.96

Note: The extra water is calculated from the water absorption of the RCA and is absorbed by the RCA and does not participate in the hydration reaction of the cement.

**Table 6:** Test results of mechanical properties.

Groups	Orthogonal Design	*f*_cu_ (MPa)	*f*_ts_ (MPa)	*f*_ts_/*f*_cu_	*f*_c_ (MPa)	*f*_c_/*f*_cu_	*E*_c_ (MPa)
SFRRC-1	A1B1C1D1	48.4	3.74	0.077	34.4	0.71	32,900
SFRRC-2	A1B2C2D2	51.9	4.46	0.086	37.4	0.72	32,700
SFRRC-3	A1B3C3D3	47.1	5.10	0.108	35.6	0.79	26,400
SFRRC-4	A2B1C2D3	39.2	4.10	0.104	28.2	0.72	32,000
SFRRC-5	A2B2C3D1	37.0	4.29	0.116	25.2	0.71	29,200
SFRRC-6	A2B3C1D2	35.1	2.87	0.082	26.8	0.73	25,400
SFRRC-7	A3B1C3D2	36.0	4.39	0.122	24.8	0.72	30,500
SFRRC-8	A3B2C1D3	33.8	2.61	0.077	24.0	0.71	27,400
SFRRC-9	A3B3C2D1	32.7	3.29	0.100	23.9	0.74	24,100

**Table 7:** Experimental data and information about prediction.

Source	No.	Compressive Strength (MPa)			Splitting Tensile Strength (MPa)			Elastic Modulus (MPa)		
		$f_{\mathrm{cu}}=91.853+106.905\left(w/c\right)+0.043r_{a}+0.737\lambda_{m}$			$f_{\mathrm{ts}}=0.267f_{cu}^{0.677}\left(1+0.371\lambda_{m}\right)\left(1-0.056r_{a}\right)$			$E_{c}=\frac{10^{5}}{2.2+34.7/f_{\mathrm{cu}}}\left(1+0.021\lambda_{m}\right)\left(1-0.211r_{a}\right)$		
		(R^2^ = 0.87)			(R^2^ = 0.97)			(R^2^ = 0.94)		
		Calculated	Tested	Calculated/Tested	Calculated	Tested	Calculated/Tested	Calculated	Tested	Calculated/Tested
Article	SFRRC-1	48.1	48.4	0.994	3.63	3.74	0.971	32,100	32,900	0.976
	SFRRC-2	47.1	51.9	0.908	4.64	4.46	1.040	31,600	32,700	0.966
	SFRRC-3	44.9	47.1	0.953	5.03	5.10	0.986	27,600	26,400	1.045
	SFRRC-4	40.9	39.2	1.043	3.89	4.10	0.949	30,800	32,000	0.963
	SFRRC-5	38.9	37.0	1.051	4.40	4.29	1.026	29,300	29,200	1.003
	SFRRC-6	38.0	35.1	1.083	2.80	2.87	0.976	24,800	25,400	0.976
	SFRRC-7	32.6	36.0	0.906	4.37	4.39	0.995	30,400	30,500	0.997
	SFRRC-8	33.0	33.8	0.976	2.81	2.61	1.077	27,700	27,400	1.011
	SFRRC-9	29.7	32.7	0.908	3.30	3.29	1.003	24,500	24,100	1.017
Literature [33]	CR0F1	54.6	45.4	1.203	—	—	—	34,100	33,000	1.033
	CR30F1	51.5	46.0	1.120	—	—	—	32,100	34,600	0.928
	CR50F1	49.8	46.8	1.064	—	—	—	30,800	38,400	0.802
	CR100F1	45.0	47.2	0.953	—	—	—	27,200	37,800	0.720
	CR50F0	49.4	44.9	1.100	—	—	—	30,100	29,700	1.010
	CR50F0.5	49.6	45.6	1.088	—	—	—	30,400	34,000	0.894
	CR50F1.5	49.9	48.1	1.037	—	—	—	31,100	41,900	0.742
	CR50F2	50.1	49.9	1.004	—	—	—	31,600	42,900	0.737
Literature [34]	N-C30R50F1	36.0	38.0	0.947	3.76	3.53	1.065	—	—	—
	N-C45R50F1	49.0	54.1	0.906	4.78	4.47	1.069	—	—	—
	N-C60R50F1	57.6	71.5	0.806	5.78	4.94	1.170	—	—	—
	N-C45R0F1	55.5	61.5	0.902	5.37	4.59	1.170	—	—	—
	N-C45R30F1	51.60	56.0	0.921	4.95	4.53	1.093	—	—	—
	N-C45R100F1	42.4	48.1	0.881	4.29	4.12	1.041	—	—	—
	N-C45R50F0	48.6	52.7	0.922	3.80	3.85	0.987	—	—	—
	N-C45R50F0.5	48.8	53.9	0.905	4.31	4.24	1.017	—	—	—
	N-C45R50F1.5	49.1	54.6	0.899	5.27	4.53	1.163	—	—	—
	N-C45R50F2.0	49.3	55.1	0.895	5.77	4.63	1.246	—	—	—
Literature [35]	REF	43.0	29.9	1.438	2.66	3.21	0.829	29,800	31,100	0.958
	RFCA	39.9	37.7	1.058	3.07	3.53	0.870	30,400	29,200	1.041
	SF-REF	43.3	34.3	1.262	3.44	3.96	0.869	31,400	30,900	1.016
	SF-RFCA	40.2	41.5	0.969	3.86	4.48	0.862	31,600	32,100	0.984
Literature [36]	C20	30.9	29.4	1.051	2.63	2.51	1.048	29,600	26,700	1.109
	C20-10K	31	30.6	1.013	2.76	2.62	1.053	30,000	27,600	1.087
	C20-20K	31	31.1	0.997	2.84	2.7	1.052	30,200	28,000	1.079
	C20-30K	31	31.5	0.984	2.92	2.85	1.025	30,400	28,300	1.074
	C20-40K	31	31.9	0.972	3.00	2.98	1.007	30,500	29,200	1.045
	C20-60K	31.1	33.2	0.937	3.19	3.15	1.013	31,000	29,800	1.040
	C20-80K	31.2	33.5	0.931	3.31	3.22	1.028	31,200	30,300	1.030
	C40	54.3	49.9	1.088	3.77	4.12	0.915	34,500	32,100	1.075
	C40-10U	54.3	52.4	1.036	4.04	4.28	0.944	35,000	33,200	1.054
	C40-20U	54.4	52.7	1.032	4.21	4.44	0.948	35,100	33,700	1.042
	C40-30U	54.5	53.9	1.011	4.42	4.59	0.963	35,400	34,100	1.038
	C40-40U	54.5	55	0.991	4.64	4.81	0.965	35,600	35,100	1.014
	C40-60U	54.6	56.1	0.973	5.01	5	1.002	35,900	35,600	1.008
	C40-80U	54.8	56.9	0.963	5.37	5.27	1.019	36,200	36,300	0.997
Literature [37]	R0F0	43	37.1	1.159	3.08	3.30	0.933	—	—	—
	R50F0	36.5	34.9	1.046	2.88	3.00	0.960	—	—	—
	R100F0	29.9	33.5	0.893	2.72	2.90	0.938	—	—	—
	R0F0/5	43.2	38.3	1.128	3.44	4.30	0.800	—	—	—
	R50F0/5	36.60	38.5	0.951	3.36	3.90	0.861	—	—	—
	R100F0/5	30.1	34.8	0.865	3.05	3.70	0.824	—	—	—
	R0F1	43.3	33.1	1.308	3.38	5.50	0.615	—	—	—
	R50F1	36.8	32.2	1.143	3.23	4.60	0.702	—	—	—
	R100F1	30.2	29.5	1.024	2.95	4.30	0.686	—	—	—
Literature [38]	R0SF0	—	—	—	—	—	—	31,700	37,100	0.854
	R30SF0	—	—	—	—	—	—	28,200	30,000	0.940
	R70SF0	—	—	—	—	—	—	24,000	21,400	1.121
	R100SF0	—	—	—	—	—	—	21,900	19,900	1.101
	R30SF1	—	—	—	—	—	—	28,900	32,200	0.898
	R70SF1	—	—	—	—	—	—	25,300	23,700	1.068
	R100SF1	—	—	—	—	—	—	22,700	20,800	1.091
	R30SF2	—	—	—	—	—	—	29,600	33,000	0.897
	R70SF2	—	—	—	—	—	—	25,900	25,100	1.032
	R100SF2	—	—	—	—	—	—	23,200	21,200	1.094
	R30SF3	—	—	—	—	—	—	30,400	34,500	0.881
	R70SF3	—	—	—	—	—	—	26,600	27,000	0.985
	R100SF3	—	—	—	—	—	—	23,900	22,100	1.081

Note: No. represents name of test specimen in the literature.

**Table 8:** Pore characteristic parameters and strength of SFRRCs.

Groups	Orthogonal Design	Total Porosity (%)	Total Pore Volume (mL/g)	Average Pore Diameter (nm)	*f*_cu_ (MPa)	*f*_ts_ (MPa)
SFRRC-1	A1B1C1D1	15.56	0.0727	32.56	48.4	3.74
SFRRC-2	A1B2C2D2	11.88	0.0591	15.88	51.9	4.46
SFRRC-3	A1B3C3D3	15.12	0.0716	30.73	47.1	5.10
SFRRC-4	A2B1C2D3	13.88	0.0712	17.32	39.2	4.10
SFRRC-5	A2B2C3D1	18.13	0.0863	30.17	37.0	4.29
SFRRC-6	A2B3C1D2	18.77	0.0993	36.61	35.1	2.87
SFRRC-7	A3B1C3D2	19.17	0.0879	33.69	36.0	4.39
SFRRC-8	A3B2C1D3	20.32	0.1031	39.81	33.8	2.61
SFRRC-9	A3B3C2D1	18.88	0.0921	19.7	32.7	3.29

## Data Availability

Not applicable.
